# Drivers, processes, and outcomes related to burnout and moral injury in the public safety workforce: a scoping review

**DOI:** 10.3389/frhs.2026.1778314

**Published:** 2026-03-31

**Authors:** Samantha A. Meeker, Margaret Ziemann, Alys Barton

**Affiliations:** 1 Department of Public Health Sciences, Parkinson School of Health Sciences and Public Health, Loyola University Chicago, Chicago, IL, United States; 2 The Fitzhugh Mullan Institute for Health Workforce Equity, Department of Health Policy and Management, Milken Institute School of Public Health, The George Washington University, Washington, DC, United States

**Keywords:** burnout, moral distress, moral injury, first responders, public safety workers, scoping review

## Abstract

**Introduction:**

Despite growing awareness of mental health, stress, and trauma among public safety workers (EMTs, firefighters, and emergency dispatchers), gaps remain in programs and research addressing burnout and moral injury -especially when compared to the attention given to healthcare professionals and, to some extent, police officers. The objective of this study is to systematically review the literature on the environmental, relational, and operational drivers, processes, and outcomes associated with burnout and moral injury among public safety personnel according to a national framework.

**Methods:**

A systematic search following PRISMA extension for Scoping Reviews guidelines was conducted across six databases for peer-reviewed publications from 2004 to 2024. Search terms focused on burnout, moral injury, and public safety professions. Reference lists from included studies and key journals were also hand-searched. Identified studies were uploaded to Covidence and screened by three reviewers using defined criteria.

**Results:**

Of nearly 13,000 articles identified, 43 were included. Only three studies examined moral injury. Most studies examined individual burnout factors (e.g., age, gender), with less attention to organizational drivers. Key drivers included operational factors (e.g., occupational stress, organizational support) and non-organizational factors (e.g., traumatic events, work-family conflict). Burnout outcomes, discussed in fewer studies, primarily related to individual-level physical and mental health or job satisfaction and turnover.

**Discussion:**

Understanding burnout and moral injury from the perspective of public safety workers is critical to public health, given their frontline role during crises. While they safeguard the well-being of others, their own health has significant implications for downstream providers and patient outcomes. Although systems-level contributors to burnout and moral injury are increasingly acknowledged in broader healthcare, this lens is less often applied to public safety workers-especially in relation to moral injury. Addressing these issues requires a deeper understanding of their origins, particularly the organizational factors that shape how burnout and moral injury manifest in this workforce. Future research must address multi-level drivers to inform more effective and targeted interventions.

## 1 Introduction

Public safety personnel (PSP) play a critical role as the first line of defense for public health and safety during an emergency. The PSP workforce includes, but is not limited to, police, firefighters, emergency medical technicians (EMTs), paramedics, correctional officers, communication officials (e.g., 911 dispatchers and operators), and search and rescue professionals. Due to the high-stress and often traumatic nature of their roles, PSP are at increased risk of post-traumatic stress disorder, depression, anxiety, substance abuse, and suicidal ideation (1).

Two distinct and increasingly recognized challenges affecting PSP are burnout and moral injury (2–4). Burnout is defined in the International Classification of Diseases (ICD-11) as “a syndrome conceptualized as resulting from chronic workplace stress that has not been successfully managed” (5). This occupational phenomenon has three key features: emotional exhaustion, depersonalization, and reduced personal accomplishment (5). Moral injury, meanwhile, was first studied in war veterans and has since been updated and researched in other occupations (6). Moral injury refers to the psychological distress that results from two aspects, betrayal and transgression (7). First, there is an act of betrayal by a trusted authority figure or authoritative body (8). Second, the betrayal, recognized or not, leads to the transgression of deep moral beliefs and socially accepted codes or laws (9). Moral distress, can be defined as the emotional response to these moral dilemmas that occurs on the continuum before moral injury (9).

An early 2021 survey of EMS personnel found that almost 40% experienced burnout. Further, a 2023 survey administered to firefighters across five states found that almost 60% of the firefighters had experienced a morally-injurious event (10). Despite a growing awareness of PSP mental health challenges, stress, trauma, burnout, and especially moral injury are comparatively less visible as occupational phenomena affecting this workforce (9). While both concepts are well-documented in healthcare professionals and, to some extent, police officers (9), there is limited research that explores these concepts among the broader non-police public safety workers. These professionals may experience different occupational stressors and organizational demands, therefore generalizing findings from police-majority samples risks overlooking profession-specific factors. Recent global events, such as the COVID-19 pandemic, have intensified the demands on PSP and demonstrated their indispensable role, as well as highlighted the urgent need to address burnout and moral injury in this workforce.

This scoping review builds on prior literature that has focused on PSP mental health or stress/trauma to examine burnout and moral injury as distinct concepts that occur along a continuum, using the National Framework for Addressing Burnout and Moral Injury in the Health and Public Safety Workforce as a guiding structure (11). While individual aspects of burnout and moral injury among public safety workers have been studied, no prior review has systematically mapped this literature using a comprehensive framework that captures their interrelated drivers, processes, and outcomes. A scoping review approach enables us to identify the extent and nature of this evidence base and assess how well existing research aligns with this newly developed framework. The aim of this review is to synthesize the evidence related to the drivers, processes, and outcomes of burnout and moral injury in public safety workers. The review is guided by the following research question: “What is known from the literature about the drivers, processes, and outcomes related to worker well-being, inclusive of moral injury and burnout, among public safety workers?”.

## 2 Methods

### 2.1 Search strategy

We conducted a systematic literature search of peer-reviewed publications from January 1, 2004 to April 30, 2024 according to the PRISMA extension for Scoping Reviews (PRISMA-ScR) guidelines. We searched six electronic databases selected for their coverage of health, emergency services, and social science literature: PubMed, Scopus, PsycINFO, Cochrane Library, SocINDEX, and Sociology Collection. We supplemented this search with a backward citation searching of reference lists of included studies from database searching and from high-impact journals in the related field (e.g., Journal of Emergency Management, Prehospital Emergency Care, Prehospital and Disaster Medicine, International Journal of Emergency Services). This search continued until no additional sources in reference lists were identified, based on title. Search terms were broken into two categories: population and phenomenon. We used the following list of terms under population: public safety worker, public safety personnel, first responder*, firefight*, emergency services, emergency medical technician*, EMT*, emergency medical services, ambulance, medics, fire and rescue, emergency medical dispatcher, EMD, dispatcher, rescue worker, fire service, and ambulance service.

The phenomenon terms focused on three domains: burnout (burnout, burned out, burn-out, emotional exhaustion, depersonali*, personal accomplishment, occupational stress), moral injury (moral injur*, morally injurious, moral distress, psychological safety), and wellness (wellness, well-being, psychological well-being, wellbeing, psychological wellbeing). The final search strings can be found in Supplementary S1.

### 2.2 Eligibility screening

Identified studies were uploaded to Covidence® and screened for eligibility using a two-stage process. In the first stage, three investigators (SM, MZ, and AB) independently screened titles and abstracts to identify studies for full-text review. Each record was reviewed by two investigators, with discrepancies resolved through discussion and, when needed, adjudication by a third investigator.

The following eligibility criteria were applied: (1) the study population included public safety workers excluding law enforcement; (2) the variable of interest included burnout (i.e., emotional exhaustion, depersonalization, personal accomplishment, occupational stress) and/or moral injury (i.e., morally injurious event, moral distress, psychological safety, psychological wellbeing); (3) occupational stress (i.e., organizational, workplace) as a variable of interest must be related to a driver of either burnout or moral injury not the outcome of the study; (4) studies with mixed populations (e.g., Medical staff and first responders) include only those that analyze first responders separately or have more than 50% of the participants as first responders; (5) full text in English; (6) US only populations; (7) studies are peer-reviewed; and (8) studies employed a methodological approach (i.e., commentaries, expert opinions, and prevalence studies were excluded).

In the second stage, the same three investigators independently reviewed the full text of all remaining studies, including those identified through hand searching, using the same eligibility criteria. Studies independently deemed eligible by two investigators were included. Disagreements were resolved through group discussion until consensus was reached on the final sample.

### 2.3 Organizing framework and data extraction

**Figure 1:**
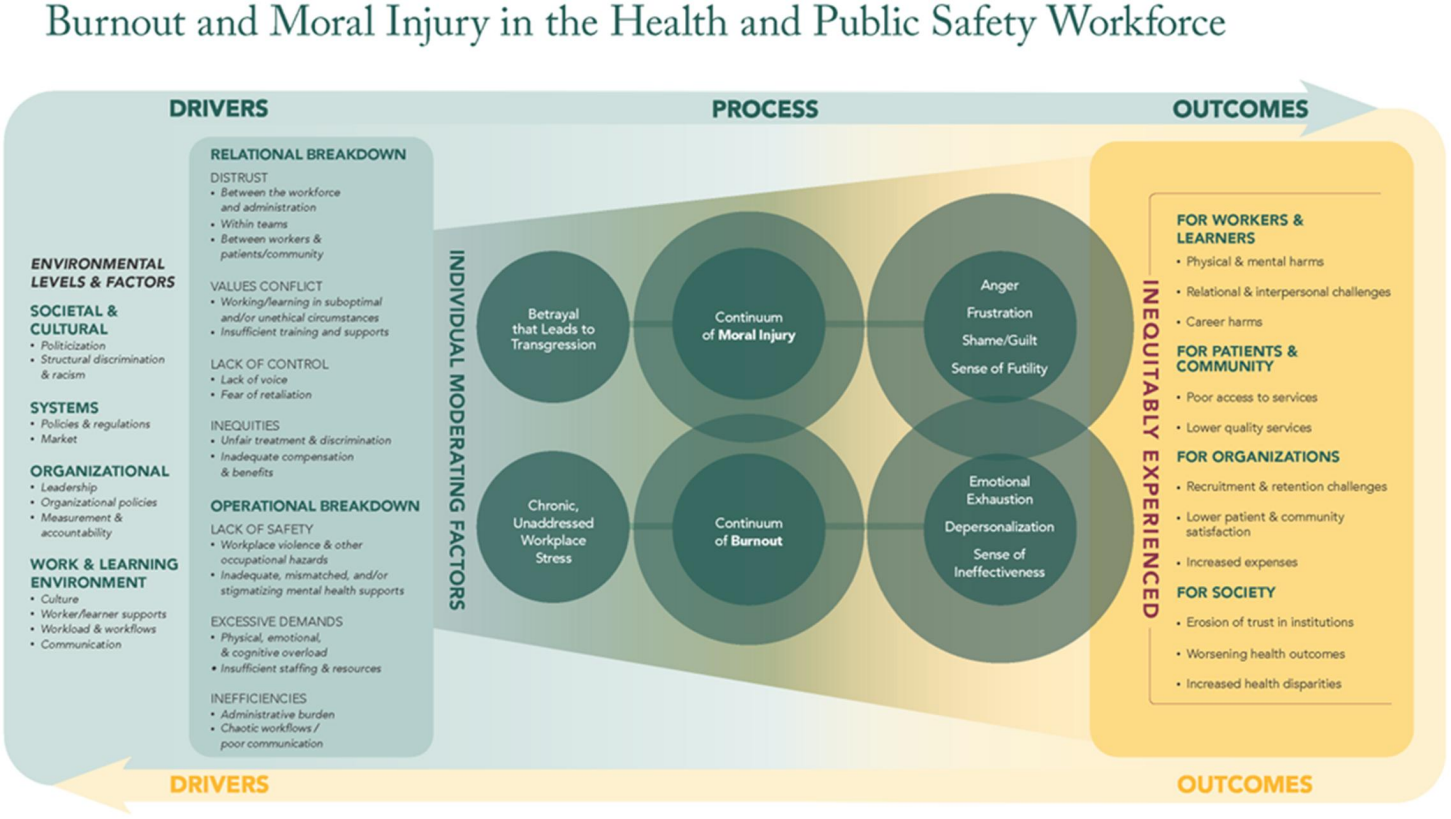
The workplace change collaborative at the Fitzhugh Mullan Institute for Health Workforce Equity; Institute for Healthcare Improvement; Moral Injury of Healthcare; AFT Healthcare. Burnout and moral injury in the health and public safety workforce (13).

To guide our assessment of the literature and extend beyond identifying the presence of burnout or moral injury among public safety personnel, we used a novel framework to structure our synthesis. The *National Framework for Addressing Burnout and Moral Injury in the Health and Public Safety Workforce* (11) (Figure 1) defines the drivers, processes, and outcomes of burnout and moral injury. Building on seminal work by Leiter and Maslach (12), the framework acknowledges multi-level environmental contributors while distinguishing between operational and relational organizational drivers. While operational drivers are more commonly addressed, relational breakdowns are often overlooked despite being central to moral injury. These occur most acutely when organizational demands conflict with patient/community care, forcing health and public safety workers to act against their professional and ethical commitments. It also recognizes the cyclical nature of burnout and moral injury in the workforce, with outcomes extending beyond the individual to impact patients, communities, organizations, and society at large.

A literature data extraction tool was developed in Covidence, using the framework (Figure 1) as an organizing structure to capture key study elements (Supplementary S2). These included general information (e.g., abstract, topic, location in US), study characteristics (e.g., method, outcome of interest, study aim, participants), results, and discussion notes. Data extraction was independently performed by two investigators for each study. The three investigators met to discuss any discrepancies in data or quality scoring and come to a consensus. Data from the relevant studies was extracted and exported to Microsoft Excel where SM performed analysis. The data were then sorted manually into the corresponding framework categories.

## 3 Results

**Figure 2:**
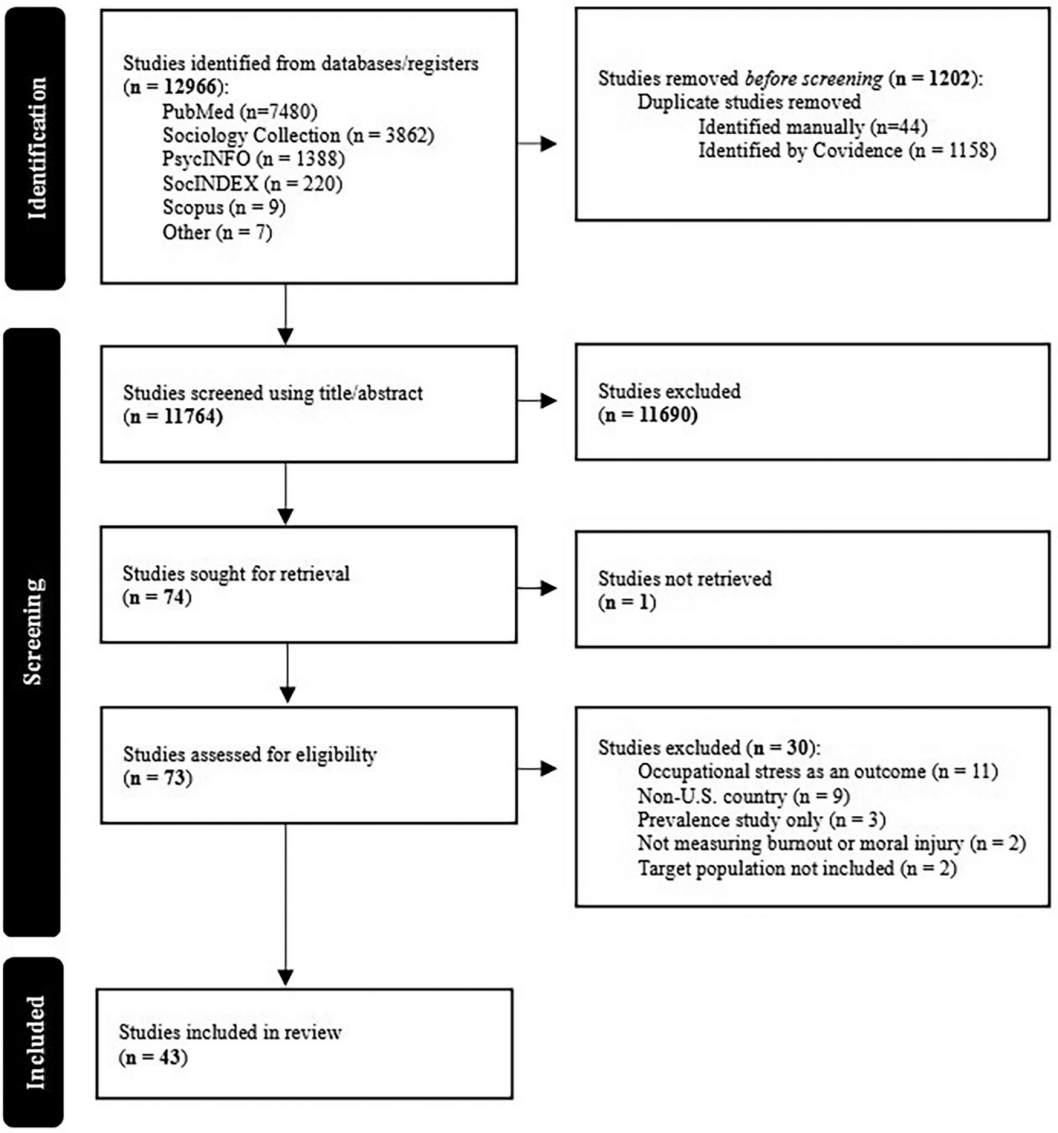
PRISMA guidelines.

This scoping review identified 43 studies examining burnout and moral injury among PSP (Table 1). A total of 11,764 individual studies were retrieved from 5 databases and through handsearching. The number was reduced to 74 after screening for title/abstract. Once the studies were assessed for eligibility, 30 were excluded, leaving 43 total studies. See Figure 2 for more information. The results are organized according to the Workplace Change Collaborative's Burnout & Moral Injury Framework, which examines three categories of drivers (relational, operational, and non-organizational), process development factors (individual characteristics, temporal progression, prevalence rates, and mediating/moderating mechanisms), and outcomes (impacts on workers and organizations). Almost all of the included studies focused on burnout (*n* = 40), with only 3 examining moral injury.

**Table 1:** Characteristics of included studies.

Author(s)	US State/region	Study design	Study aim	Study population^a^; Sample size, n	Topic	Topic measurement instrument	Methodology
Basting et al. (14)	National	Cross-sectional study	To evaluate social risks and social across EMS providers’ career and assess subgroup differences license type, sex, employment status, and burnout.	Paramedics; *n* = 1,112	Burnout	Two-question screen^b^	Descriptive; Chi-Squared
Bethea et al. (15)	West Virginia	Cross-sectional study	To examine burnout incidence, its associated factors, provider knowledge of burnout and intervention infrastructure in rural healthcare practitioners.	EMS (45%), nurses (38%), physicians (9%), advanced practice providers (nurse practitioners and physician assistants) (3%), physical therapists (1%) and other practitioners (4%); *n* = 127	Burnout	Mini Z Burnout Survey	Descriptive; T-test/Rank Testing; Chi-Squared
Blau & Chapman (16)	National	Cross-sectional study	To examine the factors contributing to EMS attrition, evaluate their importance, and assess their relationship with post-EMS life satisfaction and intentions to return to EMS.	EMS; *n* = 127	Burnout; Occupational stress	Single question on exit survey^c^	Descriptive; Correlation testing
Blau et al. (17)	National	Cross-sectional study	To examine associations between emotional labor strategies and work exhaustion across three EMS roles.	EMT-Basic (47%), EMT-Intermediate (8%), Paramedics, (45%); *n* = 24,586	Burnout	3-item scale^c^	Descriptive; Regression Modeling; Correlation testing
Boland et al. (18)	Minnesota	Cross-sectional study	To assess burnout prevalence, critical incident exposure and severity, and their associations.	EMTs/paramedics (91%) and dispatchers (9%); *n* = 209	Burnout	MBI	Descriptive; T-test/Rank Testing; Regression Modeling
Boland et al. (19)	Minnesota	Cross-sectional study	To assess burnout prevalence, coping behaviors and social connectedness, and their associations.	Paramedics (92%) and dispatchers (8%); *n* = 167	Burnout	MBI	Descriptive; Regression Modeling
Campos et al. (20)	National	Cross-sectional study	To examine EMS professionals’ experience and training in delivering adult death notifications and their association with burnout.	EMT (55%), AEMT/paramedics (45%); *n* = 1,514	Burnout	CBI	Descriptive; T-test/Rank Testing; Regression Modeling; Chi-Squared
Chiang et al. (21)	North Carolina	Cross-sectional study	To assess how autonomy influences firefighters’ stress and perceived job competence.	Firefighters; *n* = 166	Occupational Stress	17-item stress scale^b^	Descriptive; Correlation testing; Other: factor analysis
Chiang et al. (22)	South	Cross-sectional study	To examine the prevalence, occupational impact, and severity predictors of subthreshold PTSD among firefighters.	Firefighters; *n* = 164	Burnout	Compassion Fatigue-Short Scale	ANOVA; Correlation testing; Other: MANOVA
Crowe et al. (23)	National	Cross-sectional study	To examine burnout prevalence, associated characteristics, and its relationship with workforce instability among national-certified EMS professionals.	EMTs (36%) and paramedics (64%); *n* = 2,153	Burnout	19-item CBI	Descriptive; Regression Modeling
Crowe et al. (4)	South Carolina	Cross-sectional study	To assess agency-level variation in burnout and the combined influence of job resources and work-related demands on burnout among EMS professionals.	EMS; *n* = 1,271	Burnout	CBI	Descriptive; Chi-Squared; Multivariable generalized estimating equation models
Ducar et al. (24)	Virginia	Non-randomized experimental study	To evaluate the feasibility and potential effects of a mindfulness intervention on stress, mindfulness, burnout, compassion, and trauma among community EMS workers.	EMT; *n* = 15	Burnout; Occupational stress	ProQOL; PSS	Descriptive; T-test/Rank Testing; Correlation testing
Dyal et al. (25)	Southeast	Cross-sectional study	To examine whether burnout mediates the relationship between occupational stress and sleep quality and whether occupational stress mediates the relationship between sleep duration and burnout.	Firefighters; *n* = 161	Burnout; Occupational stress	10-item scale^c^; Six-item scale^c^	Regression modeling; Correlation testing; Mediation analysis
Essex & Scott (26)	New York	Cross-sectional study	To evaluate the relationship between chronic stress and coping strategies among volunteer EMS professionals.	EMS; *n* = 139	Burnout; Occupational stress	MBI-HHS	Descriptive; T-test/Rank Testing; ANOVA; Correlation testing; Chi-Squared
Folwell & Kauer (27)	Northwest	Qualitative research	To describe volunteer EMTs experience with and coping strategies for occupational stress.	Volunteer EMTs; *n* = 25	Occupational stress	Not stated.	Qualitative
Fragoso et al. (28)	Midwest	Cross-sectional study	To assess the relationship between occupational demands, personal resources, engagement, and burnout.	EMTs (55%) and paramedics (45%); *n* = 99	Burnout	CBI, OBI	Descriptive; Regression Modeling; Correlation testing
Freeman et al. (29)	National	Cross-sectional study	To examine the differences in challenges of recruiting and retaining staff between rural and urban EMS professionals.	EMS; *n* = 1,425	Burnout	Pilot-tested and revised survey^b^	Descriptive; Chi-Squared
Halbesleben (30)	Southwest	Cross-sectional study	To examine how shift type influences emotional exhaustion through work-family conflict and social support using the conservation of resources (COR) framework.	Firefighters; *n* = 168	Burnout	EE-MBI-GS.	Descriptive; T-test/Rank Testing; Regression Modeling
Hoff et al. (31)	North Carolina	Qualitative research	To examine self-reported experiences of shame and guilt and resulting emotional themes.	EMS; *n* = 8	Burnout	Not stated.	Qualitative
Kaplan et al. (32)	Pacific Northwest	Non-randomized experimental study	To examine the impact of a mindfulness-based resilience training on mindfulness, resilience, burnout, and the mediating role of resilience in the mindfulness-burnout relationship among law enforcement and firefighters.	Firefighters (32%) and law enforcement (68%); *n* = 69	Burnout	OBI	Regression Modeling; Other: nonparametric bias-corrected bootstrapping
Kaplan et al. (33)	North Carolina	Cross-sectional study	To assess the prevalence and predictors of burnout in EMS professionals.	EMTs (20%), AEMTs (7%), and paramedics (73%); *n* = 686	Burnout	ProQOL	T-test/Rank Testing; ANOVA; Regression Modeling; Correlation testing; Chi-Squared
Kaufman et al. (34)	National	Cross sectional study	To examine the co-occurrence of alcohol misuse and PTSD and associated coping mechanisms among public safety professionals.	Firefighters (25%), law enforcement (9%), paramedics/EMTs (33%), and correction officers (9%); *n* = 320	Moral Injury	Moral Injury Events Scale	Descriptive; Correlation testing; Other: Kruskal–Wallis H Tests, Dunn's Test
Knobloch & Owens (35)	Tennessee	Qualitative research	To examine public safety professionals’ experiences of moral injury, its impacts on their lives, and suggested support strategies.	Firefighter (42%), law enforcement (36%), EMS (31%), dispatch (22%), social services (14%), nonemergency medical care (11%), and corrections (6%); *n* = 36	Moral Injury	Not stated.	Qualitative
Lee et al. (36)	West Coast, Southwest, Northeast	Cohort study	To assess the influence of organizational safety climate and emotional exhaustion on safety behaviors and wellbeing over time.	EMS; *n* = 208	Burnout	5-item EE-MBI	Descriptive; Other: multilevel modeling for repeat measures
Lu et al. (37)	Washington	Cross-sectional study	To examine the prevalence and association of burnout and workplace incivility in EMS professionals.	EMS; *n* = 835	Burnout	CBI	Descriptive; Regression Modeling
McCall (38)	Southeast	Non-randomized experimental study	To examine the impact of a peer-support pilot intervention on quality of life and its association with secondary traumatic stress, burnout, and compassion satisfaction among air medical crew members.	Air medical crew; *n* = 60	Burnout	ProQOL	Descriptive; T-test/Rank Testing; ANOVA; Other: Mann–Whitney U test
McGarry & O’Connor (54)	Massachusetts	Cross-sectional study	To assess the prevalence and associated factors of burnout in EMS professionals at public and private services.	EMS; *n* = 386	Burnout	CBI	Descriptive; Other: prevalence ratios w/ 95% CI
Melnyk et al. (39)	National	Mixed-methods	To assess burnout pre- and post-pandemic in EMS professionals.	EMS; *n* = 1,882	Burnout; Occupational stress	CBI; PSS 4	Descriptive; Qualitative
Miller & Unruh (40)	Florida	Cross-sectional study	To examine compassion satisfaction, burnout, and secondary traumatic stress, and related individual and work-level factors among public safety professionals.	Law enforcement (61%), fire (20%), dispatch (14%), EMS (6%); *n* = 1,360	Burnout	ProQOL Version 5	Descriptive; Regression Modeling
Pace et al. (41)	Southwest	Cohort study	To assess the impact of an app-based mediation intervention on anxiety, depression, burnout, and negative affect in firefighters.	Firefighters; *n* = 35	Burnout; Occupational stress	10-item Burnout Measure	T-test/Rank Testing
Renkiewicz & Hubble (42)	North Carolina	Cross-sectional study	To examine potentially traumatic experiences, lifetime prevalence of suicidality, and factors related to lifetime prevalence of suicidality in EMS professionals.	EMS; *n* = 686	Burnout	ProQOL	Descriptive; T-test/Rank Testing; ANOVA; Correlation testing; Other: GLMM
Renkiewicz & Hubble (42)	North Carolina	Cross-sectional study	To assess the prevalence and predictors of compassion fatigue in EMS professionals.	EMS; *n* = 681	Burnout	ProQOL	T-test/Rank Testing; ANOVA; Regression Modeling; Chi-Squared
Renkiewicz & Hubble (43)	North Carolina	Cross-sectional study	To assess the prevalence and predictors of vicarious trauma in EMS professionals.	EMS; *n* = 691	Burnout	ProQOL	T-test/Rank Testing; Regression Modeling
Roth et al. (44)	National and Canada	Cross-sectional study	To assess the roles of moral injury and emotion regulation in the relationship between adverse childhood experiences (ACEs) and mental health symptoms in public safety professionals.	Firefighter (19%), paramedic (44%), law enforcement (17%), dispatcher (6%), and PSP-other (14%); *n* = 249	Moral Injury	MIA-PSP	Descriptive; Regression Modeling; Correlation testing; Other: Hayes’ PROCESS macro (2018), model 7
Sattler et al. (45)	Washington	Cross-sectional study	To examine factors associated with stress and posttraumatic growth among firefighters within COR and posttraumatic growth theoretical frameworks.	Firefighters; *n* = 286	Burnout; Occupational stress	57-item scale^b^	Descriptive; Regression Modeling; Correlation testing
Schwartz et al. (46)	Northeast	Cross-sectional study	To examine the moderating role of empathy and gender on the association between occupational stress and mental health in EMS professionals.	EMS; *n* = 568	Burnout; Occupational stress	Mini Z Burnout Survey (1-item); SOOS-14	Descriptive; T-test/Rank Testing; Regression Modeling; Chi-Squared
Sliter et al. (47)	Midwest	Cohort study	To examine the relationships between traumatic occupation stressors and PTSD, burnout, and absenteeism in firefighters.	Firefighters; *n* = 179	Burnout; Occupational stress	OBI; Impact of Events Scale	Descriptive; Regression Modeling
Smith et al. (48)	Southeast	Cross-sectional study	To examine the relationships between occupational stress, work-family conflict, burnout, and safety behaviors in firefighters.	Firefighters; *n* = 208	Burnout; Occupational stress	10-item scale from^c^; 6-item scale^c^	Descriptive; Regression Modeling; Correlation testing; Other: path analysis
Smith et al. (49)	Southeast	Cross-sectional study	To examine the relationship between work pressure, perceived stress, and work-family conflict and burnout in firefighters.	Firefighters; *n* = 208	Burnout	10-item scale^c^	Descriptive; Regression Modeling; Correlation testing
Smith et al. (50)	East and West	Cross-sectional study	To examine the relationships between occupational stress, burnout, and safety behaviors in firefighters.	Firefighters; *n* = 742	Burnout; Occupational stress	10-item scale^c^; 6-item scale^c^	Descriptive; Correlation testing; Other: Structural Equation Modeling
Stout et al. (51)	Colorado	Cross-sectional study	To examine the relationship between wildfire response and burnout, compassion fatigue, and vicarious trauma in wildlife firefighters.	Wildlife firefighters; *n* = 186	Burnout	MBI-GS; Compassion Fatigue Self-Test	Descriptive; T-test/Rank Testing; ANOVA; Other: MANOVA
Witkowski et al. (52)	National	Cross-sectional study	To assess whether work pressure, workplace support strategies, and COVID-related strategies are associated with substance use and whether burnout mediates this association in public safety professionals.	Fire (40%), EMS (36%), law enforcement (25%); *n* = 2,801	Burnout	EE-MBI	Descriptive; Correlation testing; Other: Structural Equation Modeling
Wolkow et al. (53)	National	Cross-sectional study	To examine the association between sleep disorder risk and mental health outcomes and burnout in addition to the mediating role of sleep at work in firefighters.	Firefighters; *n* = 6,307	Burnout	MBI	Descriptive; ANOVA; Regression Modeling

AEMT, advanced emergency medical technician; CBI, Copenhagen Burnout Inventory; EE-MBI, emotional exhaustion subscale; EMS, emergency medical services; EMT, emergency medical technician; IES, Impact of Events Scale; MBI, Maslach Burnout Inventory; MBI-GS, Maslach Burnout Inventory–General Survey; MBI-HSS, Maslach Burnout Inventory for Health and Human Services; MIA-PSP, Moral Injury Assessment for Public Safety Personnel; OBI, Oldenburg Burnout Inventory; ProQOL, Professional Quality of Life Scale 5; PSS, Perceived Stress Scale; PTSD, post-traumatic stress disorder; SOOS, Sources of Occupational Stress.

^a^ Study population profession percentages do not sum to 100% due to rounding and self-reporting serving in multiple capacities or not reporting.

^b^ Study-specific novel instrument.

^c^ Instrument adapted from cited articles in study.

While a full quality assessment was not completed as this is a scoping review, the studies were nearly all cross-sectional with small sample sizes. The average number of participants was 1,383 with a range from 8 to 24,586% and 81% of the studies utilized cross-sectional designs. Due to the methodological constraints of cross-sectional studies and the small sample sizes, it was often difficult to determine if the samples were truly representative of the population of interest. The geographic scope of individual studies was often limited, and while organizational contexts were described, inter-organizational comparisons and analyses accounting for organizational clustering were largely absent, which constrained the strength of the evidence provided.

### 3.1 Drivers

Twenty-one studies examined drivers of burnout among PSP; none examined drivers of moral injury (Table 2). This scoping review assessed drivers using three categories: relational, operational, and non-organizational.

**Table 2:** Drivers and outcomes of burnout and moral injury examined in included studies.

Domain	Category	Factors	Components	Direction of relationship	Article(s)
Drivers	Relational drivers	Values conflict	Lack of workplace support	↑BO^a^	McCall (38); McGarry & O'Connor (54); Miller & Unruh (40); Sattler et al. (45); Witkowski et al. (52)
			Poor alignment with employer values	↑BO^a^	Bethea et al. (15)
			Limited access to employee-sponsored debriefings	↑BO^a^	Kaplan et al. (33)
		Lack of control	Lack of control over workload	↑BO^a^	Bethea et al. (15)
			Lack of perceived autonomy	↑OS^a^	Chiang et al. (21)
		Distrust	Incivility	↑BO^a^	Lu et al. (37)
		Inequities	Inadequate compensation	↑BO^a^	McGarry & O'Connor (54); Witkowski et al. (52)
			Benefits (i.e., time off)	↓BO^a^	Witkowski et al. (52)
	Operational drivers	Lack of safety	Sleep disruption	↑BO^a^	Wolkow et al. (53)
			Unsafe work practices	↑BO^a^	Smith et al. (48); McGarry & O'Connor (54)
			Incompetent coworkers	↑OS^a^	Folwell & Kauer (27)
		Excessive demands	General job demands	↑BO^a^	Crowe et al. (4); Fragoso et al. (28); Halbesleben (30); Witkowski et al. (52)
			Patient-facing workload	↓BO^a^^,^^c^	Schwartz et al. (46)
			Call volume	↑BO^a^	Crowe et al. (23); McGarry & O'Connor (54)
			Inadequate time to process trauma	↑BO^a^	McGarry & O'Connor (54)
		Inefficiencies	Time pressures	↑BO^a^	Crowe et al. (4)
			Lack of access to decompression spaces	↑BO^a^	Witkowski et al. (52)
			Role ambiguity and role efficiency	↑BO^a^	Bethea et al. (15)
				↑EE^a^	Halbesleben (30)
			Staff shortage	↑BO^a^	Folwell & Kauer (27)
			Documentation	↑BO^a^	Bethea et al. (15)
		Employment characteristics	Paid vs. volunteer	↑BO^a^^,^^b^	Blau et al. (17); Miller & Unruh (40); Sattler et al. (45)
			Private vs. fire-based agency	↑BO^a^	Crowe et al. (23)
	Non-organizational drivers	Exposure to traumatic events	General exposure to critical incident/traumatic event	↑BO^a^	Sattler et al. (45); Sliter et al. (47)
			Recurrent high stress calls	↑BO^a^	Folwell & Kauer (27)
			Delivering death notifications	↑BO^a^	Campos et al. (20)
			Vicarious trauma	↑BO^a^	Kaplan et al. (33)
			Compassion fatigue	↑BO^a^	Kaplan et al. (33)
		Patient interactions	Knowing patients personally	↑OS^a^	Folwell & Kauer (27)
			Lack of gratitude from patients	↑BO^a^	McGarry & O'Connor (54)
			Patient abuse	↑BO^a^	McGarry & O'Connor (54)
		Work-life balance	Work-family or work-life conflict or	↑BO^a^	Fragoso et al. (28); Halbesleben (30); Smith et al. (49); Smith et al. (48)
				↑OS^a^	
Outcomes	Worker-level outcomes	Mental health	Depression	↑BO^a^	Fragoso et al. (28)
				↑EE^a^	Lee et al. (36)
				↑OS^a^	Schwartz et al. (46)
			PTSD	↑BO^a^	Sattler et al. (45)
				↑MI^a^	Kaufman et al. (34)
			Substance use	↑BO^a^	Chiang et al. (22); Witkowski et al. (52)
				↑MI^b^	Kaufman et al. (34)
			Distress	↑BO^a^	Chiang et al. (22)
				↑MI^a^	Knobloch & Owens (35)
			Other mental health outcomes	↑BO^a^	Blau & Chapman (16); Renkiewicz & Hubble (55); Renkiewicz & Hubble (42); Renkiewicz & Hubble (43); Schwartz et al. (46); Lee et al. (36); Chiang et al. (22)
				↑MI^a^	Knobloch & Owens (35)
		Physical health	General health	↑BO^a^	Fragoso et al. (28)
				↑EE^a^	Blau et al. (17)
			Sleep quality and duration	↑BO^a^	Dyal et al. (25)
		Social functioning	Social isolation and suspicion of others	↑MI^a^	Knobloch & Owens (35)
	Organizational-level outcomes	Job satisfaction and attrition	Leaving EMS	↑BO^a^	Blau & Chapman (16); Crowe et al. (23); Freeman et al. (29)
			Turnover intention	↑BO^a^	Fragoso et al. (28)
			Likelihood of using sick days	↑BO^a^	Crowe et al. (23)
		Job performance	Workplace conflict	↑BO^a^	Melnyk et al. (39)
			Workplace safety	↑BO^a^	Smith et al. (50)
				↑EE^a^	Lee et al. (36)

BO, burnout; EE, emotional exhaustion; MI, moral injury; OS, occupational stress.

^a^ Measure of association.

^b^ Measure of correlation.

^c^ Schwartz et al. (46) found that increased workload was especially associated with decreased burnout in women.

#### 3.1.1 Relational drivers

The relational drivers section of the Burnout & Moral Injury Framework comprises four distinct categories: distrust, values conflict, lack of control, inequities, and distrust. The majority of studies did not examine relational drivers of burnout and moral injury. Among the nine studies that did, seven examined values conflict factors, two considered lack of control, two considered inequities, and one investigated distrust.

##### 3.1.1.1 Values conflict

Values conflict was operationalized through three primary themes: workplace support, alignment with employer values, and access to debriefings. Five studies examined workplace support and consistently found that absence of coworker and managerial support—whether formal or informal—was associated with increased burnout risk (38, 40, 45, 52, 54). One study found that lack of experience with or knowledge of incidence debriefing resources was associated with higher burnout (33). Additionally, poor values alignment with one's employer was associated with higher levels of burnout (15).

##### 3.1.1.2 Lack of control

Two studies examined lack of control as a relational driver. Bethea et al. (15), found that lack of control over workload was associated with burnout, while Chiang et al. (21) found that lower perceived autonomy support was associated with higher occupational stress levels, a known precursor to burnout.

##### 3.1.1.3 Inequities

Two studies considered compensation which falls under inequities in the framework: McGarry and O'Connor (54) found that inadequate compensation was associated with increased burnout, while Witkowski et al. (52) found that being compensated when required to quarantine was also associated with an increase in burnout (however the study believed that the extra pay didn't cause burnout but instead may have been related to the nuances of the COVID-19 quarantine measures), while being able to take time off was associated with a decrease in burnout. Finally, Lu et al. (37) found that experiencing workplace incivility was associated with higher levels of burnout.

#### 3.1.2 Operational drivers

The operational drivers section of the framework comprises three distinct categories: lack of safety, excessive demands, and inefficiencies. A total of 15 studies examined operational drivers. Of these, three studies addressed lack of safety, eight examined excessive demands, and five investigated inefficiencies. An additional seven studies identified operational factors that did not align with the existing framework categories.

##### 3.1.2.1 Lack of safety

Three studies examined different dimensions of safety in relation to burnout. Wolkow et al. (53) found that insufficient sleep due to overnight shifts was associated with higher degrees of burnout. Smith et al. (48) found that both adherence to safe work practices and personal protective equipment compliance were associated with lower burnout rates. McGarry and O'Connor (54) found that a history of work-related injury was associated with increased burnout risk. Additionally, Folwell and Kauer (27) identified concerns about the competency of fellow EMTs as one of four major occupational stressors among staff.

##### 3.1.2.2 Excessive demands

Seven studies examined excessive demands through various operational factors. Four studies considered general job demands and three found they were associated with increased odds of burnout (4, 28, 30). However, one notable exception found that as workloads increased—particularly patient-facing work—burnout actually decreased, especially among women (46).

Two studies examined call volume specifically. Crowe et al. (23). found that exceeding 20 calls per week was positively associated with burnout, while McGarry and O'Connor (54) found that inadequate breaks between calls contributed to burnout. McGarry and O'Connor (54) also found that inadequate time to process trauma was associated with higher burnout levels. Finally, Witkowski et al. (52) found that greater work pressure was positively associated with burnout.

##### 3.1.2.3 Inefficiencies

Five studies examined inefficiencies in the work environment. Crowe et al. (4) identified multiple efficiency-related factors: time pressures were associated with a four-fold increase in burnout odds, waiting for emergency calls in a vehicle rather than at the base was associated with a two-fold increase in burnout odds, and lacking access to facilities for eating, food preparation, showering, storage, exercise, or relaxation at work was associated with increased burnout odds. Similarly, Witkowski et al. (52) found that absence of decompression spaces was associated with increased burnout.

Bethea et al. (15) found that both a chaotic work atmosphere and lower perception of efficient teamwork were associated with higher burnout. Bethea et al. (15) also found that both unsatisfactory time for documentation and excessive time spent on documentation were significantly associated with burnout. In a qualitative study, Folwell and Kauer (27) found that staff attributed burnout to inadequate personnel, noting that “lacking personnel causes stress because of increased work-load, worry, anxiety, and burnout.” Halbesleben (30) found that role ambiguity and role conflict were associated with emotional exhaustion.

##### 3.1.2.4 Other operational factors

Three studies identified organizational benefits associated with decreased burnout: mental health services (52), job resources (4), and access to continuing education (20).

##### 3.1.2.5 Employment characteristics

Studies examining volunteer vs. paid employment status yielded mixed findings. Blau et al. (17) found that working as a volunteer increased emotional exhaustion, while Miller and Unruh (40) found volunteer status was associated with lower burnout, and Sattler et al. (45) found it was correlated with lower burnout. And finally, working as an EMT at a private agency vs. a fire-based agency increased the odds of burnout (23).

#### 3.1.3 Non-organizational drivers

Several burnout drivers were related to work but fell outside organizational control. These included exposure to trauma and critical incidents, patient interactions, and work-life balance.

##### 3.1.3.1 Exposure to trauma and critical incidents

Five studies examined the relationship between exposure to traumatic events and burnout. Delivering a greater number of death notifications (20), recurrent highly stressful calls (27), and exposure to critical incidence/traumatic events (45, 47) were all associated with an increase in burnout. Additionally, vicarious trauma and compassion fatigue related to work were associated with increased burnout (33).

##### 3.1.3.2 Patient interactions

Two studies identified patient-related factors associated with burnout. Folwell and Kauer (27) found that knowing patients personally—such as treating members of one's own community—was associated with increased stress. McGarry and O'Connor (54) found that lack of gratitude from patients and patient abuse were both associated with burnout.

##### 3.1.3.3 Work-Life balance

Four studies found that work-life conflict or work-family conflict increased stress and burnout (28, 30, 48, 49).

### 3.2 Process development

The process development component of the Workplace Change Collaborative's Framework examines how individual characteristics, temporal factors, and intervening mechanisms influence the development and progression of these conditions. Thirty-two of the studies examined the process of developing burnout or moral injury among PSP (Table 3). This section considers individual characteristics (recognizing that personal experiences and social determinants are carried into the workplace and affect experiences with burnout and moral injury), temporal progression, prevalence rates, and mediating or moderating factors.

**Table 3:** Process development of burnout and moral injury examined in included studies.

Domain	Factors	Components	Direction of relationship	Article(s)
Individual characteristics	Demographics	Sex	↑BO^a^^,^^b^	Crowe et al. (23)^c^; Sattler et al. (45)^c^; Lu et al. (37)^d^
			↓PA^b^^,^^e^	Essex & Scott (26)
			↓EE^b^^,^^e^	Essex & Scott (26)
			No significant relationship	Kaplan et al. (33); McCall (38); Miller & Unruh (40); Schwartz et al. (46)
		Race/ethnicity	↑BO^b^	Kaplan et al. (33)^f^
			↓BO^b^	Miller & Unruh (40)^g^
			No significant relationship	Lu et al. (37); Schwartz et al. (46); Smith et al. (48)
		Age	No significant relationship	Kaplan et al. (33); McGarry & O'Connor (54); Miller & Unruh (40); Smith et al. (48)
		Education level	No significant relationship	Kaplan et al. (33); Miller & Unruh (40); Smith et al. (48)
		Marital status	No significant relationship	Kaplan et al. (33); McCall (38); Miller & Unruh (40); Schwartz et al. (46); Smith et al. (48)
		Sexual orientation	No significant relationship	Kaplan et al. (33)
	Psychological and behavioral	Surface acting	↑EE^b^	Blau et al. (17)
		Controlled emotional regulation	↑OS^b^	Chiang et al. (21)
		Nonreactivity	↑BO^b^	Kaplan et al. (32)
		Resilience	↓BO^b^	Miller & Unruh (40); Schwartz et al. (46)
	Adverse experiences and social determinants	ACEs	↑BO^b^	Kaplan et al. (33)
			↑MI^b^	Roth et al. (44)
		Housing/food insecurity, healthcare inaccessibility, social isolation, sleep disorders, mental health problems, and extensive sense of duty	↑BO^b^	Basting et al. (14); Boland et al. (19); Wolkow et al. (53); Folwell & Kauer (27)
	Coping mechanisms	General coping strategies	↓OS^b^	Folwell & Kauer (27)
		Social support	↓BO^b^	Boland et al. (19); Essex & Scott (26)
			↓MI^b^	Knobloch & Owens (35)
		Mindfulness	↓BO^b^	Kaplan et al. (32); Pace et al. (41); Ducar et al. (24)^h^
		Substance use	↑BO^b^	Basting et al. (14); Boland et al. (19); Essex & Scott (26)
		Self-blame	↑BO^b^	Boland et al. (19)
		Food	↑BO^b^	Boland et al. (19)
		Doing the bare minimum	↑BO^b^	Essex & Scott (26)
		Looking forward to off-duty time	↑BO^b^	Essex & Scott (26)
Temporal progression	Years of experience	More years of experience	↑BO^b^	Blau et al. (17); Chiang et al. (21); Crowe et al. (23); Kaplan et al. (33); Lu et al. (37); Sattler et al. (45); Stout et al. (51)
			No significant relationship	Essex & Scott (26); McGarry & O'Connor (54); Schwartz et al. (46)
		Loss of passion	↑BO^b^	Hoff et al. (31)
Prevalence rates	Self-reported moral injury in PSP	Experienced by all participants.	-	Knobloch & Owens (35)
	Screened positive for burnout in EMS	16 to 87.7%, 37.2% median	-	Basting et al. (14); Bethea et al. (15); Boland et al. (18); Boland et al. (19); Kaplan et al. (33); Lu et al. (37); McGarry & O'Connor (54); Schwartz et al. (46)
	High burnout in at least one of the three burnout dimensions (PA, EE, DP) in firefighters	48.1%	-	Wolkow et al. (53)
	Burnout by EMS agency	20% no burnout, 8% all EMS professionals experienced burnout, 35% median burnout level	-	Crowe et al. (4)
	Burnout by type (personal, work-related, and patient-related) in paramedics and EMTs	Personal: 38.3% of paramedics and 24.9% of EMTs; Work-related: 30.1% of paramedics and 19.1% of EMTs; Patient-related: 14.4% of paramedics and 5.5% of EMTs	-	Crowe et al. (23)
	Work exhaustion by profession	45% of EMT-basics, 39% of EMT-intermediates, and 49% of paramedics		Blau et al. (17)
Mediating and moderating factors	Moderating factors	Level of emotional regulation difficulties	ACEs → MI	Roth et al. (44)
		Humor	Traumatic stress → BO	
	Mediating factors	BO	Workplace support strategies → substance abuse	Wolkow et al. (53)
		BO	OS → sleep quality	Dyal et al. (25)
		OS	BO → sleep duration	Dyal et al. (25)
		Sleep quality	Sleep → BO; Mental health → BO	Wolkow et al. (53)
		Perceived competence	Autonomous regulation → OS	Chiang et al. (21)
Potential progression pathway	Experiencing poor patient outcomes	Feeling deficient leading to feeling shame	↑BO	Hoff et al. (31)

ACEs, adverse childhood experiences; BO, burnout; DP, depersonalization; EE, emotional exhaustion; EMS, emergency medical services; EMT, emergency medical technician; MI, moral injury; OS, occupational stress; PA, personal accomplishment; PSP, public safety professionals.

^a^ Measure of association.

^b^ Measure of correlation.

^c^ Crowe et al. (23) and Sattler et al. (45) found being male was associated or correlated with higher burnout.

^d^ Lu et al. (37) found women were more likely to experience burnout than men.

^e^ Essex & Scott (26) found that being female was associated with decreased burnout subscales—personal accomplishment and emotional exhaustion.

^f^ Kaplan et al. (33) found participants self-identified as white or “two or more races” had the highest rates of burnout.

^g^ Miller & Unruh (40) found that Hispanic participants had lower burnout than white participants.

^h^ Ducar et al. (24) found that participation in a mindfulness intervention significantly reduced burnout.

Six studies examined the relationship between occupational stress and burnout, consistently finding that increased occupational stress was associated with increased burnout (15, 28, 46, 48–50). Three study found that low job satisfaction was associated with increased burnout (17). Two studies found that burnout was negatively associated with job satisfaction: Chiang et al. (22) found this relationship for burnout in general, while Blau et al. (17) found it specifically for emotional exhaustion.

#### 3.2.1 Individual characteristics

Individual characteristics were the most extensively studied area in this review, with 22 studies examining this domain. Of these, 19 focused on various individual characteristics, while 8 examined coping mechanisms either alone or in conjunction with individual characteristics.

##### 3.2.1.1 Demographic characteristics

Sex was the most commonly examined demographic variable; however, all studies restricted measurement to binary sex categories (male and female), and none reported data on gender identity or gender-diverse populations. Findings were mixed: two studies found that being male was associated with or correlated with higher levels of burnout (23, 45). Essex and Scott (26) found that being female was associated with lower scores on both the personal accomplishment and emotional exhaustion scales, while Lu et al. (37) found that women were more likely to experience burnout than men. Witkowski et al. (52) found that being female was correlated with higher burnout in bivariate analysis, but this relationship was no longer significant in a structural model. Four studies found no significant association between sex and burnout (33, 38, 40, 46).

Race was examined in five studies with inconsistent findings, as well. Kaplan et al. (33) found that EMS professionals who identified as white or as “two or more races” had the highest rates of burnout. Miller and Unruh (40) found that Hispanic dispatchers had lower levels of burnout than white dispatchers. Three studies found no significant relationship between race and burnout (37, 46, 48).

Age was examined by four studies, all of which found it was not a significant characteristic (33, 40, 48, 54). Similarly, education was examined by three studies, none of which found it to be significant (33, 40, 48). Marital status was examined by five studies, all finding no significant association with burnout (33, 38, 40, 46, 48) Sexual orientation was examined by one study, which also found no significant association (33).

##### 3.2.1.2 Psychological and behavioral characteristics

Three studies examined emotional reactivity and regulation. Blau et al. (17) found that surface acting—displaying emotions that are not genuinely felt—was positively associated with emotional exhaustion. Chiang et al. (21) found that higher levels of controlled regulation (a focus on external pressures such as avoiding letting colleagues down or impressing others) was associated with increased occupational stress. Conversely, Kaplan et al. (32) found that nonreactivity, a component of mindfulness, was associated with decreased burnout.

Two studies examined resilience, both finding that increased resilience was negatively associated with burnout (40, 46).

##### 3.2.1.3 Adverse experiences and social determinants

Two studies examined adverse childhood experiences (ACEs). Kaplan et al. (33) found that ACEs were positively associated with burnout, while Roth et al. (44) found they were positively associated with moral injury.

Several studies identified social determinants and health characteristics associated with burnout. Basting et al. (13) found that housing insecurity, food insecurity, and healthcare inaccessibility were all positively associated with burnout. Individual studies also found associations between burnout and social isolation (19), sleep disorders (53), and mental health problems (53). One qualitative study found that participants identified their extensive sense of duty as a source of stress (27).

##### 3.2.1.4 Coping mechanisms

Seven studies examined coping mechanisms in relation to burnout, and one examined them in relation to moral injury. Social support emerged as a protective characteristic: two studies found it was negatively associated with burnout (19, 26), while one found it to be negatively associated with moral injury (35).

Mindfulness consistently demonstrated protective effects. Two studies found that mindfulness was associated with lower burnout (32, 41), and one intervention study found that mindfulness training statistically significantly reduced burnout (24).

Maladaptive coping strategies were associated with increased burnout. Three studies found that substance use was positively related to burnout (13, 19, 26). Individual studies also found positive associations between burnout and self-blame (19), using food to cope (19), doing the bare minimum at work (26), and focusing on looking forward to off-duty time (26). One qualitative study found that both formal and informal coping strategies can be effective at minimizing stress related to job duties (27).

#### 3.2.2 Temporal progression

To understand temporal progression of burnout and moral injury, we examined years of experience in the field. No studies examined temporal progression for moral injury specifically. Seven studies generally found that more years of experience was associated with higher burnout (17, 21, 23, 33, 37, 45, 51), while 3 found no association (26, 46, 54). The impact of years of experience may be related to loss of passion, which was found to be associated with increased burnout in one study (31).

##### 3.2.2.1 Prevalence rates

Although studies were not selected based on whether they reported prevalence rates, many included this information. One qualitative study examined moral injury and reported that all participants had experienced moral injury in their work as first responders (35).

Twelve studies reported burnout rates, though their measurement methods varied considerably. Among studies that reported the percentage of participants screening positive for burnout, rates among EMS workers ranged from 16% to 87.7% (13, 15, 18, 19, 33, 37, 46, 54), with a median of 37.2% across the eight studies.

Wolkow et al. (53) examined burnout dimensions among firefighters and found that 48.1% exhibited high burnout in at least one of three dimensions: personal accomplishment, emotional exhaustion, or depersonalization. Crowe et al. (4) examined rates by agency and found that 20% of EMS agencies reported no burnout, 8% reported that all EMS professionals were experiencing burnout, and the median burnout level across all agencies was 35%.

Crowe et al. (23) distinguished between three types of burnout (personal, work-related, and patient-related) and found that 38.3% of paramedics and 24.9% of EMTs met criteria for personal burnout; 30.1% of paramedics and 19.1% of EMTs met criteria for work-related burnout; and 14.4% of paramedics and 5.5% of EMTs met criteria for patient-related burnout. Blau et al. (17) examined only work exhaustion and found that 45% of EMT-basics, 39% of EMT-intermediates, and 49% of paramedics experienced work exhaustion.

#### 3.2.3 Mediating and moderating factors

Eight studies examined mediating or moderating factors: one for moral injury and seven for burnout. It should be noted that we retained the specific terminology (mediating vs. moderating) as reported in each original paper, as authors made these distinctions based on their theoretical frameworks and statistical analyses.

For moral injury, Roth et al. (44) found that the relationship between ACEs and moral injury symptoms was moderated by the level of emotional regulation difficulties.

For burnout, several mediating and moderating relationships were identified. Witkowski et al. (52) found that burnout mediated the relationship between workplace support strategies and problematic substance abuse. Wolkow et al. (53) found that the association between sleep and mental health problems with increased burnout in firefighters was mediated by sleep quality during overnight work.

Humor functioned as a moderating factor: those low in coping humor showed a stronger relationship between traumatic stressors and burnout (47). Kaplan et al. (32) found that both mindfulness and nonreactivity were mediating factors that led to increased resilience and therefore decreased burnout.

Dyal et al. (25) identified bidirectional mediation relationships: the relationship between occupational stress and sleep quality was fully mediated by burnout, while the relationship between burnout and sleep duration was fully mediated by occupational stress. Chiang et al. (21) found that perceived competence mediated the relationship between autonomous regulation and stress, such that autonomous regulation increased perceived competence, which in turn decreased stress.

Finally, one qualitative study identified a potential progression pathway: when EMS clinicians experienced poor patient outcomes and felt deficient, this could lead to feelings of shame, which often progressed to feelings of burnout (31).

### 3.3 Outcomes

Eighteen studies examined outcomes related to burnout and moral injury. These outcomes were categorized as impacts on workers (*n* = 14) or impacts on organizations (*n* = 7). No studies examined patient or community-level impacts.

#### 3.3.1 Worker outcomes

##### 3.3.1.1 Physical health impacts

Three studies examined physical health outcomes associated with burnout and moral injury. Two studies found that burnout negatively affected general health: work exhaustion was negatively associated with physical health (17), and burnout was negatively associated with overall worker health (28). One study found that burnout was negatively associated with both sleep quality and sleep duration (25).

##### 3.3.1.2 Mental health impacts

Twelve studies examined mental health outcomes. Depression was examined by three studies: occupational stress was associated with increased odds of depression (46), emotional exhaustion was associated with depression (36), and burnout was associated with depressive symptoms (28).

Three studies examined substance use. Kaufman et al. (34) found that alcohol use was positively correlated with moral injury, while two studies found that substance use in general was positively associated with burnout (22, 52).

Two studies examined post-traumatic stress disorder (PTSD). Moral injury was positively associated with PTSD in one study (34), while burnout was positively associated with PTSD in another (45). Two studies examined distress: burnout was associated with greater general distress (22), and moral injury was associated with spiritual distress (35).

Individual studies identified positive associations between burnout and multiple additional mental health outcomes, including life satisfaction after leaving EMS (16), suicidality (42), compassion fatigue (55), vicarious trauma (43), occupational stress (46), and anxiety (46). Burnout was negatively associated with morale (36) and self-efficacy (22), while moral injury was negatively associated with self-esteem (35).

##### 3.3.1.3 Social functioning impacts

One qualitative study examined the impact of moral injury on social functioning and found that participants reported moral injury led to social isolation and suspicion of others (35).

#### 3.3.2 Organizational outcomes

Seven studies examined organizational outcomes of burnout, focusing on job satisfaction and attrition (*n* = 4) and job performance (*n* = 3).

##### 3.3.2.1 Attrition

Three studies examined actual turnover and turnover intentions. Blau and Chapman (16) found that burnout was the most important factor in leaving EMS jobs and was negatively associated with returning to the field. Crowe et al. (23) found that burnout increased both the likelihood of leaving the job or profession and the likelihood of using 10 or more sick days in the past year. Fragoso et al. (28) found that burnout was associated with turnover intention. One study found that rural agencies were more likely to lose staff due to burnout compared to urban agencies (29).

##### 3.3.2.2 Job performance

Three studies examined how burnout affects job performance. Two studies found that burnout negatively impacted workplace safety: Lee et al. (36) found that emotional exhaustion negatively affected safety compliance behaviors, while Smith et al. (50) found that burnout was negatively associated with multiple safety behaviors including safety citizenship behavior, safe work practices, personal protective equipment use, and safety compliance. One study found that burnout was associated with increased workplace conflicts, including short tempers and poor working relationships (39).

## 4 Discussion

This scoping review explored what is known from the extant literature on the drivers, processes, and outcomes associated with burnout and moral injury among PSP, using a novel framework inclusive of both occupational phenomena as an organizing structure for our synthesis.

We found the vast majority of studies meeting inclusion criteria focused on burnout, with only 3 focused on moral injury—a finding consistent with a gap in the evidence base identified in a prior systematic review (9). This limited attention to moral injury stands in stark contrast to the frequency with which public safety workers are exposed to potentially morally injurious events, including acts of commission, omission, or witnessing harm that violates deeply held moral values (56). While not all such exposures result in moral injury, the lack of empirical inquiry into its origins, mechanisms, and consequences represents a critical evidence gap that constrains both prevention and intervention efforts.

Among the studies examining the drivers of burnout, the majority focused on those that were operational in nature such as workload, shift work, and staffing levels. Fewer than one quarter of studies examined relational drivers, and none examined the role of trust explicitly, despite the inherently relational nature of public safety work. Public safety workers engage continuously with colleagues, supervisors, patients, families, and community members in high-stakes contexts where interpersonal dynamics can profoundly shape wellbeing (52). National data suggest that nearly half of emergency medical services workers, for example, do not feel valued by their employer or sufficiently recognized for their performance, while about one-third do not think their agency fosters teamwork and camaraderie (57). These experiences are deeply relational in nature and closely aligned with work-life domains of reward, fairness, and community described by Leiter and Maslach in their seminal work on burnout (12).

While operational drivers undoubtedly contribute to chronic workplace stress and subsequent burnout among public safety workers, the relative absence of relational and trust-based determinants in the literature likely obscures key mechanisms affecting workers' well-being and through which moral injury, specifically, develops and persists. Moreover, most of the studies in our review examining the burnout and moral injury process focused on individual-level factors such as worker demographics and lived experience. This emphasis reinforces a persistent orientation toward individual-level explanations for worker distress, while the potential contributions of organizational and system-level factors, such as governance structures, funding policies, staffing models, labor practices, and interagency coordination, remain largely unexamined, despite the fact that public safety workers routinely operate within complex, interconnected systems spanning law enforcement, healthcare, and social services.

Similarly, of the included studies that examined outcomes associated with moral injury and burnout, nearly all focused on the individual worker-level, with the majority finding associations between burnout, its precursors, or moral injury and poor mental health outcomes like depression, anxiety and PTSD. This line of inquiry is critical for a workforce that often encounters traumatic events, operates in high-stress environments, and for which more firefighters and EMS personnel have historically died by suicide than in the line of duty, and it reinforces prior calls for improved surveillance of mental health conditions to inform targeted interventions (58, 59). Equally notable, however, is the absence of any community or patient/consumer-level outcomes associated with burnout and moral injury among the studies we reviewed. This narrow focus on individual worker outcomes may inadvertently weaken policy urgency by failing to demonstrate how burnout and moral injury reverberate beyond the workforce to undermine public safety, care quality, and population health.

Although examining the effectiveness of interventions was outside the scope of this review, the high and persistent prevalence of burnout and the emerging recognition of moral injury signal an urgent need for action. The studies included in our review provide some evidence for protective factors that may be considered in the design of strategies to improve public safety well-being and prevent burnout and moral injury. These include managerial, peer and social support, worker autonomy, adequate time off, access to continuing education, mindfulness, and humor. However, the evidence supporting these strategies should be interpreted in light of the study designs, as findings were often derived from cross-sectional survey data and cannot establish causality. These strategies build upon broader trends in public safety wellbeing intervention research, which often prioritize resilience training, stress management, or self-care while paying comparatively little attention to the structural conditions that produce chronic strain (60). Yet individual or operational fixes—while necessary—are alone unlikely to resolve what is increasingly understood as a systemic problem in the caring professions (61).

Public safety workforce challenges make these findings especially urgent. Shortages are growing across sectors (62), and some agencies, most visibly in law enforcement (63, 64), have relaxed entry standards. This trend could extend to other public safety professions facing recruitment and retention challenges. At the same time, the Bureau of Labor Statistics projects faster than average growth for public safety professions like EMTS and paramedics through 2034 (65)– demand driven by expected retirements and converging with national trends of increased climate-related disasters, emerging public health threats, strained health systems, and eroding trust in government. Burnout and moral injury further weaken workforce capacity, performance, and retention, creating a destabilizing feedback loop. Addressing these issues requires improved measurement and reporting, greater awareness of relational and organizational factors, and stronger policy action. Some states have begun taking meaningful steps, offering early examples of legislative and regulatory approaches that could be applied more broadly (66). Monitoring the effectiveness of such policy actions will be essential for informing long-term systems change.

### 4.1 Limitations

As with all research, this scoping review has several limitations that must be considered. First, while we conducted hand searches of high-impact journals and reference lists, we did not include a comprehensive grey literature search of conference proceedings, dissertations, government reports, or organizational white papers, which may have contained additional relevant research or emerging findings not yet published in peer-reviewed journals. This may have introduced publication bias, as studies with null or negative findings are less likely to appear in published literature.

Second, due to the limited number of studies identified, we could not analyze individual types of public safety personnel (e.g., firefighters, paramedics, emergency dispatchers) separately, which may obscure important differences in burnout and moral injury experiences, risk factors, and protective factors across these distinct occupational groups. Our inclusion criteria also required studies with mixed populations to either analyze first responders separately or include more than 50% of first responders. This threshold, while necessary for maintaining focus, may have excluded studies with valuable insights from samples with slightly lower proportions of public safety personnel.

Third, the strength of the evidence base was constrained by the methodological characteristics of the included studies. The literature was dominated by cross-sectional designs, often with limited geographic scope and relatively small or non-representative samples, which restricts inference about temporal relationships, causality, and generalizability to the broader public safety workforce. Additionally, although organizational context was frequently described, few studies incorporated analytic approaches that accounted for organizational clustering or other multi-level influences (e.g., unit-, agency-, or system-level factors). These gaps underscore the need for more robust research using nationally representative public safety workforce data, longitudinal designs, and multilevel analytic approaches that can better capture how individual, organizational, and structural factors interact to shape burnout and moral injury over time.

Finally, the heterogeneity in how burnout and moral injury were measured and operationalized across studies may limit direct comparisons. Different assessment tools, definitions, and conceptualizations of these constructs were employed across the literature, which is inherent to scoping reviews but should be considered when interpreting findings.

## 5 Conclusion

Ultimately, protecting public health depends on protecting the wellbeing of the workforce that safeguards it. Taken together, our findings highlight the need to move beyond an individualistic framing of burnout and moral injury toward a systems-oriented research and policy agenda for addressing PSP wellbeing. Future studies should prioritize: (1) deeper examination of the organizational, relational, and structural origins of burnout and moral injury; (2) rigorous evaluation of multi-level interventions that target governance, staffing, interagency coordination, and workplace culture; and (3) explicit assessment of downstream impacts on patient care, community wellbeing, and institutional trust. Without such a shift, efforts to strengthen and stabilize the public safety workforce will remain fragmented and insufficient to meet the growing threats to public health and safety. Improved measurement and reporting of both burnout and moral injury inclusive of instruments capable of capturing relational and system-level determinants will be essential to raising awareness, strengthening causal inference, and driving policy response.

## Data Availability

The original contributions presented in the study are included in the article/Supplementary Material, further inquiries can be directed to the corresponding author.
