# Supplementary material for: Drivers, processes, and outcomes related to burnout and moral injury in the public safety workforce: a scoping review
Source: Front Health Serv. 2026 Mar 31;6:1778314. doi: 10.3389/frhs.2026.1778314 (PMC13076284; doi:10.3389/frhs.2026.1778314)
Supplement: Supplementary file 1 [file Datasheet1.pdf]

Supplementary Data

1. Search terms

- Block 1: Population
  (“public safety worker*” OR “public safety personnel” “first responder*”, OR firefight* OR “emergency medical personnel” OR EMS OR paramedic* OR “search and rescue*” OR “emergency services” OR “emergency medical technician*”OR EMT* OR “emergency medical services” OR ambulance, medics OR “fire and rescue” OR “emergency medical dispatcher” OR EMD OR dispatcher OR “rescue worker” OR “fire service” OR “ambulance service”)
- Block 2: Phenomenon - Burnout
  (burnout OR "burned out" OR burn-out OR "burnt out" OR "emotional exhaustion" OR depersonali* OR "personal accomplishment" OR “occupational stress”)
- Block 2: Phenomenon – Moral Injury
  ("moral injur*" OR “morally injurious” OR "moral distress" OR "psychological safety")
- Block 2: Phenomenon – Wellness
  (Wellness OR "well-being" OR "psychological well-being" OR wellbeing OR “psychological wellbeing”)
- Combined Terms
  (“public safety worker*” OR “public safety personnel” “first responder*”, OR firefight* OR “emergency medical personnel” OR EMS OR paramedic* OR “search and rescue*” OR “emergency services” OR “emergency medical technician*”OR EMT* OR “emergency medical services” OR ambulance, medics OR “fire and rescue” OR “emergency medical dispatcher” OR EMD OR dispatcher OR “rescue worker” OR “fire service” OR “ambulance service”) AND (burnout OR "burned out" OR burn-out OR "burnt out" OR "emotional exhaustion" OR depersonali* OR "personal accomplishment" OR “occupational stress”)

  (“public safety worker*” OR “public safety personnel” “first responder*”, OR firefight* OR “emergency medical personnel” OR EMS OR paramedic* OR “search and rescue*” OR “emergency services” OR “emergency medical technician*”OR EMT* OR “emergency medical services” OR ambulance, medics OR “fire and rescue” OR “emergency medical dispatcher” OR EMD OR dispatcher OR “rescue worker” OR “fire service” OR “ambulance service”) AND ("moral injur*" OR “morally injurious” OR "moral distress" OR "psychological safety")

  (“public safety worker*” OR “public safety personnel” “first responder*”, OR firefight* OR “emergency medical personnel” OR EMS OR paramedic* OR “search and rescue*” OR “emergency services” OR “emergency medical technician*”OR EMT* OR “emergency medical services” OR ambulance, medics OR “fire and rescue” OR “emergency medical dispatcher” OR EMD OR dispatcher OR “rescue worker” OR “fire service” OR “ambulance service”) AND (Wellness OR "well-being" OR "psychological well-being" OR wellbeing OR “psychological wellbeing”)

2. Covidence data extraction

General information

1. Title
2. Author(s)
3. Year
4. Possible conflicts of interest for study author(s)
5. Abstract
6. Location in U.S.
7. Topic
    - i. Burnout
    - ii. Moral injury
    - iii. Occupational stress
8. Notes

Characteristics of included studies

1. Methods
    - a. Aim of study
    - b. Outcome of interest
    - c. Study design
        - i. Randomized controlled trial
        - ii. Non-randomized experimental study
        - iii. Cohort study
        - iv. Cross-sectional study
        - v. Case-control study
        - vi. Qualitative research
        - vii. Prevalence study
        - viii. Case series
        - ix. Case report
        - x. Other
    - d. Burnout instrument
    - e. Moral injury instrument
    - f. Occupational stress instrument
    - g. Other instruments
    - h. Start date
    - i. End date
2. Participants
    - a. Population description
    - b. Inclusion criteria
    - c. Exclusion criteria
    - d. Method of recruitment for participants
    - e. Total number of participants
    - f. Demographic population characteristics

3. Analysis method
    i. Descriptive
    ii. T-test/Rank testing
    iii. ANOVA
    iv. Regression modeling
    v. Correlation testing
    vi. Chi-squared
    vii. Qualitative
    viii. Other

Results

1. Positive results
2. Negative results
3. Nonsignificant results
4. Qualitative results
5. Mediating and moderating factors
6. Other results

Discussion

1. Notes on discussion as necessary
2. Limitations
